# Assessment of Eating Disorders in Adolescents: Tunisian cross-sectional study

**DOI:** 10.1192/j.eurpsy.2025.1154

**Published:** 2025-08-26

**Authors:** Z. Nesrine, D. Ben Touhemie, I. Hadjkacem, J. Boudabous, K. Chiha, H. Ayédi, K. Khemekhem, Y. Moalla

**Affiliations:** 1 pédopsychiatrie, Hedi Chaker hospital, Sfax, Tunisia

## Abstract

**Introduction:**

Adolescence, around the time of puberty, is the life stage most prone to the emergence of eating disorders, with bodily and psychological transformations at the center of the issue. The body serves as a key medium for expressing distress in adolescents.

**Objectives:**

To estimate the prevalence of eating disorders (ED) among adolescents and to determine associated factors to ED

**Methods:**

This is a cross-sectional, descriptive, and analytical study conducted between June 2024 and September 2024 among adolescents resident at Sfax, Tunisia. Data were collected via an online Google Forms questionnaire exploring sociodemographic and relational data. We used the “Eating Attitudes Test 40 (EAT-40)” to detect the presence of ED, with a score of ≥ 30 indicating the presence of an eating disorder.

**Results:**

We collected data from 120 adolescents, with an average age of 16 years and a predominance of females (69.2%). The majority of participants (84.6%) lived in urban areas, 80.8% were enrolled in secondary schools, and 33.3% of adolescent have reported relational difficulties with peers. 41,5% of Adolescents were living in particular family situations( death of one parent, parental separation, relational difficulties with parents).

In our study, 30% of adolescents have a psychiatric disorder. The average total score on the EAT-40 scale was 35.03, the prevalence of eating disorders was estimated at 47.5%. The factors correlated with ED included: female gender (p=0.012), relational difficulties with peers (p=0.002), the death of one parent (p=0.024), and personal psychiatric history (p=0.00).

**Conclusions:**

This study reveals a significant prevalence of eating disorders among adolescents in Sfax, Tunisia. Gaining a deeper understanding of this condition and its underlying factors could greatly improve the care and support provided to these young individuals.Bas du formulaire

**Disclosure of Interest:**

None Declared

